# The Role of Age-Friendly Environment between Cognitive Ability and Attitude towards Gerontechnology

**DOI:** 10.1093/geroni/igab046.2492

**Published:** 2021-12-17

**Authors:** Inhye Jung, Miseon Kang, Si Young Song, Yun Mook Lim, Hey Jung Jun

**Affiliations:** Yonsei University, Seoul, Seoul-t'ukpyolsi, Republic of Korea

## Abstract

This study examined the impact of the age-friendly environment on the association between cognitive ability and the attitude towards gerontechnology. The participants were 277 Korean young-old age 65 through 74. The data were collected by an online survey conducted in February 2021. The dependent variable was the attitude towards gerontechnology sum of perceived usefulness, perceived ease of use, and perceived adaptability (Heerink et al., 2010; Xu et al., 2015). The independent variable was the cognitive ability of participants to report on their own. The moderating variable was the age-friendly environment sum of physical, service-side, and cultural conditions. Multiple regression analysis was conducted to determine the relation between cognitive ability and the attitude towards gerontechnology. The moderating effect was then analyzed using PROCESS macro and bootstrapping. Results show that cognitive ability has a positive effect on the attitude towards gerontechnology, and the age-friendly environment has softened its effectiveness. When participants were living in a more age-friendly environment, their attitude toward gerontechnology was less affected by their cognitive ability. However, when age-friendly environmental condition scores above 62 (out of 75), the environmental aspect did not affect the association between cognitive ability and attitudes to gerontechnology. This study suggests that the age-friendly environment can narrow the disparity of the attitude towards gerontechnology depending on the cognitive capability levels under certain conditions. Regarding the attitude towards technology may affect the actual use, the possibility of environmental help is meaningful.

